# The association between bullying victimization and suicidal ideation among students in Africa: a systematic review and meta-analysis

**DOI:** 10.3389/fpubh.2025.1556211

**Published:** 2025-09-09

**Authors:** Anmut Endalkachew Bezie, Lamrot Yohannes, Asmare Asrat Yirdaw, Mihretu Tagesse Sergindo, Biniam Belete Begena, Awoke keleb

**Affiliations:** ^1^Department of Occupational Health and Safety, College of Medicine and Health Sciences, Wollo University, Dessie, Ethiopia; ^2^Department of Environmental and Occupational Health and Safety, Institute of Public Health, College of Medicine and Health Sciences, University of Gondar, Gondar, Ethiopia; ^3^Department of Environmental Health, College of Medicine and Health Sciences, Arba Minch University, Arba Minch, Ethiopia; ^4^School of Public Health, Wachemo University, Hosanna, Ethiopia; ^5^Department of Public Health, Arsi University, Arsi, Ethiopia; ^6^Department of Environmental Health, College of Medicine and Health Science, Wollo University, Dessie, Ethiopia

**Keywords:** bullying, bullying victimization, suicidal ideation, suicide, students, systematic review and meta-analysis, Africa

## Abstract

**Introduction:**

Bullying victimization through cyberbullying, verbal threats, insults, or nicknames, and physically, like stealing or exclusion from the peer group, is a significant challenge for schools and teachers in educational settings. It is a precursor for suicidal ideation and has an adverse effect on students’ mental health. Therefore, we performed a systematic review and meta-analysis to investigate the association between bullying victimization and suicidal ideation among students in Africa.

**Methods:**

A thorough search of literature was conducted through international electronic databases to identify relevant studies on the association between bullying victimization and suicidal ideation. Studies published up to October, 2024 were included. The recommended PRISMA guideline was used for reporting items for this systematic review and meta-analysis. To extract and analyze data, Microsoft Excel 16 and STATA 17 software were used, respectively. The quality of the included studies was examined using the Joanna Briggs Institute critical appraisal checklist. The funnel plot and Egger’s regression test were applied to evaluate publication bias. To estimate the pooled association and heterogeneity, a random effects model and I^2^ test statistics were used, respectively.

**Results:**

This study included 16 articles that met the inclusion criteria, encompassing a total sample size of 34,007. Students with bullying victimization were 1.7-fold more prone to suicidal ideation than their counterparts (OR = 1.68, 95% CI: 1.56–1.81, I^2^ = 60.9%, *p* < 0.001). Subgroup analyses demonstrate that moderate but slightly higher heterogeneity was found among secondary school students than tertiary students and moderate heterogeneity but a high odds ratio among tertiary students. In addition, higher heterogeneity was found from primary studies than that of studies using secondary data analysis. Sensitivity analyses confirmed the robustness of these findings.

**Conclusion:**

The findings of this study demonstrated that students with bullying victimization were at higher risk of suicidal ideation than their counterparts who were not victimized. Therefore, interventions focused at school-based anti-bullying programs, counseling services, parental involvement, and improving social and peer supports were advised to mitigate the effect of bullying and reduce suicidal ideation.

**Systematic Review Registration:**

https://www.crd.york.ac.uk/PROSPERO/view/CRD42024615422

## Introduction

Suicidal ideation, which is defined as thoughts about engaging in behavior intended to end one’s life, is a significant public health problem among school adolescents (1). Although suicide is a worldwide public health problem that causes a death for 727,000 people globally (2), these deaths often begin with unrecognized or untreated suicidal thoughts. In the USA, suicide is the tenth leading cause of death and responsible for over 48,000 deaths every year (3). In China and Australia, about 250,000 (4) and 3,000 people (5) die by suicide annually, respectively. In Europe, suicide claims the lives of over 150,000 people each year and is the leading cause of death for youth (6). A systematic review on the global burden of disease showed that suicide is the 12th leading cause of death among adolescents aged 10–24 in Sub-Saharan Africa (7). In addition, according to the World Health Organization report (2018), nearly 800,000 individuals die by suicide globally each year, and it is the second cause of death for individuals aged 15–19 in low- and middle-income nations (8).

The above figures showed that early identification of suicidal ideation is a key strategy for early warning signs and preventing suicide. Suicide ideation is a precursor to suicide attempts or completed suicide and is vital for early identification and intervention (9). In an African context, suicidal ideation is an alarming and growing public health issue among students that requires urgent attention and comprehensive understanding (10–26).

Bullying victimization is another public health issue described as intentional, recurring, or prolonged exposure to harmful behaviors carried out by an individual or group of individuals who are thought to be stronger or of higher status than the victim (27, 28). Bullying can occur through cyberbullying, verbal threats, insults, or nicknames, and physical acts like stealing or exclusion from the peer group (29). It presents a serious challenge for school teachers in both public and private educational settings. The school setting is a crucial social space where students are expected to grow both intellectually and personally (30). Adolescence, marked by significant social and cognitive development is a crucial period. However, disruptions during this time can lead to negative psychosocial outcomes (31, 32). The episode of bullying during this stage can have detrimental, long-lasting effects on their mental health and well-being. This includes fear, reduced academic performance, dropout, difficulties in school adjustment (33), school absenteeism, and suicide among the young population (34–37).

Bullying has a negative impact on school safety, and students are more prone to feeling insecure in circumstances where it occurs. Episodes and acts of bullying are most often invisible to teaching staff. This makes it difficult to recognize the codes, languages, signs, and practices through which students bully or harass each other (38).

The magnitude of bullying victimization is high globally. For example, in Latin America, 37.8% of adolescents were victimized by bullying (39). Another study across 16 Latin American nations found that 46.7% of students were robbed, 35.7% were insulted or threatened, 38.9% were struck, and 62.4% witnessed for whatever type of bullying incident (38). In China the magnitude of bullying victimization was 30% (40), and the worldwide occurrence of bullying in low- and middle-income nations was 34.2% (41), with significantly higher rates reported in African countries (41–43). Another study in 19 low- and middle-income nations of middle school students reported that those who were bullied in the previous month were more prone to report feelings of despair and hopelessness, loneliness, sleeplessness, and suicidal ideation than nonbullied students (41). In addition, a study finding on the school-based health survey in 83 countries indicates that the occurrence of bullying was 35.3% (42). Moreover, previous findings have demonstrated that bullying victimization is linked with higher suicidal risk among young adults (44). Adolescents who were cyber (45) and traditional bullied victims (46) were more prone to suicidal ideation than their counterparts (46). Studies in Argentina, Panama, St. Vincent, and the Grenadines showed that 38.4% of students who faced cyberbullying victimization were more affected by suicidal ideation than those who did not face it (45).

A meta-analysis of studies also demonstrates that bullying victimization was positively linked with suicidal thought among adolescents (47, 48). The occurrence of this association was highest in the Western Pacific (42) and lowest in Scandinavian countries due to the strongest nationwide implementation and sustainability of successful bullying policies and programming (49).

Parental monitoring and connectedness with family (50), peer support (51, 52), and perceived social support buffered and lowered levels of association between victimization and suicidal thought (53). The growing corpus of research elaborates that demographics and behavioral factors such as age, gender, cigarette smoking, drug and tobacco use, physical activity, loneliness, food insecurity, peer support, and parental support were significantly related to suicidal ideation (45). Higher degrees of bullying victimization had a greater impact on suicidal thoughts in women than in men. Parent support was more effective for girls in reducing suicidal ideation, while higher parent support was more successful for men in reducing bullying victimization and suicidal thought (3).

Literature indicates females who are victims of bullying have a greater suicide risk (54), and other studies demonstrate that boys who have a greater chance of bullying victimization are more affected by suicidal ideation (55). However, the strength of association decreased when controlling for individual-level variables (56). The existing primary studies in African countries evidenced that bullying victimization is a major risk factor for suicidal ideation (10, 16–18, 23, 24, 57–64). This high degree of being bullied among teenagers in African countries could be attributed to the low socioeconomic situation of students, poor school, family, and social conditions, political violence, war, and criminality (65, 66).

The effect of bullying ranges from physical symptoms, including stomach aches, backaches, headaches, and dizziness, to bad temper, feeling nervous, difficulties getting to sleep (33), morning tiredness, feeling left out, loneliness, helplessness (67), and post-traumatic stress disorder (68). Students who have been bullied in school achieve much worse math and reading scores than those who have not. Furthermore, witnessing a classmate’s bullying has a bad impact on their academic performance (38). Bullying victimization was linked to a considerably higher risk of current smoking, alcohol and tobacco usage, truancy, physical fighting, and unprotected sexual activity (39).

In addition, bullied students may develop both internalized and externalized behaviors such as social exclusion, shame (69), aggressive behaviors (70), anxiety, low self-esteem, loneliness (71, 72), and depression, a well-known risk factor for suicide (28, 73). Indeed, they internalized suicidal ideation or externalized suicidal attempts, physical aggression, and alcohol and tobacco use behaviors (74). Moreover, to cope with the overwhelming stress and trauma of bullying victimization, students might adopt maladaptive coping strategies such as substance abuse and self-harm and engage in sexual activity (41). Furthermore, bullying affects students’ academic achievement by impairing concentration, lowering self-esteem, and exacerbating emotions of failure and hopelessness (6). This ongoing emotional upheaval can affect their normal psychological functioning and make it difficult for kids to deal with everyday challenges.

Suicidal ideation is a strong predictor of suicide. At the same time, bullying victimization is linked with suicidal thoughts. However, despite the association between bullying victimization and suicidal ideation gaining global attention, it has not received adequate attention on the African continent. Most existing studies focus on individual countries or are the outcome of global analyses. This hides or underestimates the synthesis evidence in Africa. Furthermore, despite bullying and suicidal ideation being well studied, few studies have found the pooled association between bullying victimization and suicidal ideation among students in Africa. Therefore, this systematic review and meta-analysis focuses on finding the pooled association of suicidal ideation and bullying victimization among students in Africa. The findings have important implications for the development of targeted mental health interventions and policies that can benefit students’ well-being and inspire educators and programmers to collaborate and to create safer and more encouraging learning environments.

## Methods

### Registration and reporting system

This study aimed to synthesis the existing studies that focused on the association between bullying victimization and suicidal ideation. This review adhered to the core principles outlined in the Centre for Reviews and Dissemination’s (CRD) guidance for healthcare reviews. The review protocol was registered in Prospero in November 2024 with a registration ID of CRD42024615422. This review followed the recommended reporting items of Preferred Reporting Items for Systematic Reviews and Meta-Analyses (PRISMA) 2020 guidelines.

### Data sources, study period, searching strategies, and study selection

A comprehensive literature search was performed across international electronic databases, including PubMed, Google Scholar, Science Direct, and HINARI. We incorporated studies published from the initiation of these databases up to October 20, 2024, by two authors independently (AEB, AK). We contacted subject-matter experts to gather more information on published research. To ensure a comprehensive search, we carefully reviewed the references in selected studies to identify any related studies that may have been missed. Indeed, we used a search term of prevalence of suicidal ideation and associated factors and then selectively choose the factor bullying or bullying victimization. The MeSH terms and search filters were incorporated into the search strategy using the PMC Advanced Search Builder. Boolean operators were applied to ensure comprehensive retrieval of relevant articles. The search included the following key terms: bullying OR “peer victimization” OR “school violence” OR “cyber bullying” AND suicide OR “suicidal thoughts” OR “Suicidal Ideation” OR “suicidal behavior” AND Student* OR adolescent* OR youth OR “school children” AND Africa OR “Sub-Saharan Africa” OR Algeria OR Benin OR Botswana OR “Burkina Faso” OR Burundi OR Egypt OR “Equatorial Guinea” OR Eritrea OR Eswatini OR Ethiopia OR Ghana OR Kenya OR Liberia OR Mali OR Morocco OR Mozambique OR Namibia OR Nigeria OR Rwanda OR Senegal OR “Sierra Leone” OR Somalia OR “South Africa” OR Sudan OR Tanzania OR Tunisia OR Uganda OR Zambia OR Zimbabwe (Supplementary File 1). Beyond the primary keywords, we employed synonyms, abbreviated symbols, and additional free-text keywords to enhance the search. The review covered full-text articles that were exclusively published in English. The reference lists of selected articles were manually examined, and the “similar articles” feature of the databases was utilized to identify additional relevant studies. The screening process began with an independent review of titles and abstracts, followed by a full-text screening of the selected studies by four authors (AEB, AK, LY, AAY). Any differences of opinion were settled by consensus. The selection process was meticulously documented to enable the completion of a PRISMA 2020 flow diagram.

### Eligibility criteria

#### Inclusion

Literature that fulfilled the following criteria was considered in this review. Population: The review includes students in different school levels, such as primary school, secondary school, college, and university students. Outcome variables: In this review, the outcome variable was finding the pooled association between bullying victimization and suicidal ideation. Study design: This review includes cross-sectional studies. Study setting: This review was conducted in Africa. Language: This review includes studies published in English language. Publication period: literature published up to October 20, 2024, was incorporated.

#### Exclusion criteria

Exclusion criteria were applied to studies that did not report on suicidal ideation or reported on suicidal attempts and unrelated research and studies where the full text was not available or could not be retrieved. Duplicate data sources and studies with poor methodological quality were excluded. To ensure consistency and methodological rigor, studies reporting adjusted odds ratios for bullying victimization and suicidal ideation were included in a meta-analysis, and findings reporting relative risk ratios were excluded and included qualitatively in the discussion part. Qualitative and unpublished studies, editorial letters, and non-research articles were also excluded. Moreover, studies conducted other than student populations were excluded. These criteria guarantee the review is targeted on high-quality, relevant studies that accurately assess the association between suicidal ideation and bullying victimization.

#### Data extraction and quality assessment

After all articles were exported into EndNote 20, duplicate entries were removed. The remaining data were extracted through a standardized form, which was initially piloted on two included studies. This is implemented in a Microsoft Excel 2016 form that captured study characteristics and outcomes. Four authors (AEB, MTS, BBB, AK) were responsible for extracting data from cross-sectional studies, recording details such as the authorship, publication year, school type, study design, sample size, timing of outcome measurement, outcome assessment, and odds ratio for the association between suicidal ideation and bullying victimization. Following the screening of relevant articles for eligibility by the four reviewers, the quality of each study was examined using the Joanna Briggs Institute critical appraisal checklist (75). The checklist has eight evaluation criteria with a response option of yes, no, unclear, and not applicable. Each reviewer independently evaluated the risk of bias for the studies, with the results expressed on a 100% scale. Finally, studies with low risks of biases were considered in this review. To resolve any differences that arose during the quality assessment, the mean score from all reviewers was calculated.

#### Risk of publication bias assessment

To evaluate publication bias and the potential effect of small studies, we employed Begg’s funnel plots and Egger’s test. First, we visually examined the symmetry of the funnel plots. Second, we quantitatively evaluated the likelihood of publication bias using Egger’s regression test.

#### Data analysis and synthesis

All analyses were conducted using Stata version 17. To evaluate the association between suicidal ideation and bullying victimization, we employed random effects models since the included studies represent a random sample of all possible study results. The I^2^ statistic was used to examine heterogeneity among the incorporated studies, with I^2^ values below 50% indicating homogeneity and values greater than or equal to 50% indicating presence of heterogeneity. We considered a 95% confidence interval and a *p*-value of less than 0.05 as statistically significant for associations, absence of publication bias, and heterogeneity. We used the DerSimonian-Laird estimator with the Knapp and Hartung adjustment for standard errors. To investigate potential sources of heterogeneity, subgroup analyses focusing on school type (secondary school, both primary and secondary school, and university/college) was conducted. All analyses were done using the “metan” package in the Stata software version 17.^1^

## Results

### Searching process

**Figure 1:**
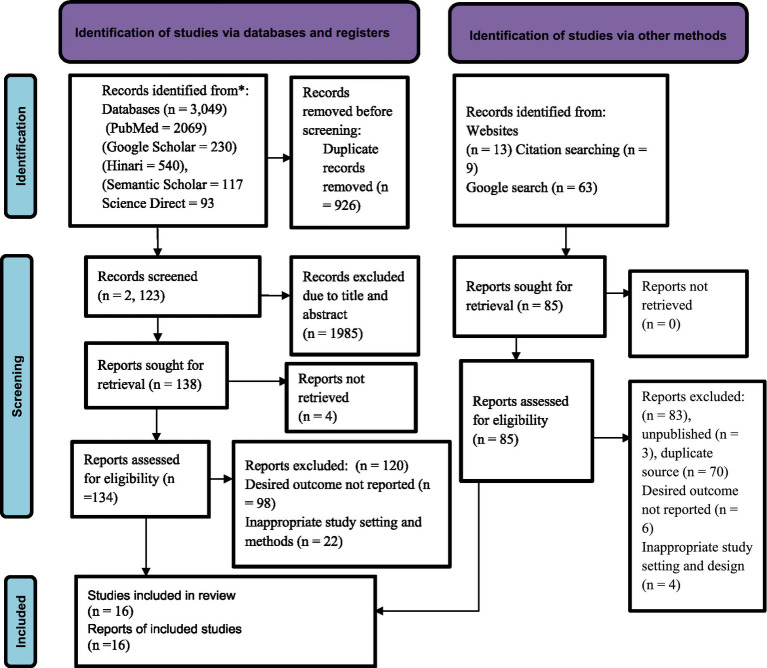
PRISMA flow diagram for the systematic review and meta-analysis of the association of bullying victimization and suicidal ideation among students in Africa, 2024.

A total of 3,134 articles were identified using electronic databases and manual searching. After deleting duplicate records from electronic databases 2, 123 articles were screened for this review. Based on their titles and abstracts, 1,985 articles were excluded. In addition, 120 articles were excluded based on the exclusion criteria. Finally, a total of 16 articles were included in this review. The PRISMA flow diagram was used to summarize the selection process (Figure 1).

### Characteristics of the included studies

In this review, we extracted the publication year, country; study design, sample size, and school type. By design, all of the included studies were cross-sectional. This review included a total of 34,007 participants (10, 16–18, 23, 24, 57–64, 76, 77). The included articles were published between 2007 and 2024, with sample sizes ranging from 400 to 3,793. In this review, two studies were conducted in each of Liberia and Uganda, five studies in Ghana, and one study was conducted in each of the following countries: Ethiopia, Eswatini, South Africa, Namibia, Mali, Tanzania, and Sierra Leone. Of the research, twelve were done with secondary or high school students; one study was conducted in primary and high school, secondary and university students and another one study was conducted in each college and university. Among the included studies, the high odds ratio for bullying victimization and suicidal ideation was found among university students (63), and the smallest odds ratio was found among rural school-going students in Ghana (58). Except for four studies (23, 58, 61, 77), 12 studies (10, 16–18, 24, 57, 59, 60, 62–64, 76) with adjusted odds ratios demonstrate that bullying victimization was significantly associated with suicidal ideation. In this systematic review, studies use various screening techniques to evaluate suicidal ideation. Standardized questionnaires were widely used, but there was no clinical confirmation or longitudinal research in any of the investigations. Some studies used the WHO Composite International Diagnostic Interview to assess suicidal ideation; ten studies used the Global School-Based Student Health Survey that was collected before and used cluster sampling techniques to select their study participants. The majority of the studies use self-administered questionnaires (Table 1). Table 1 provides a summary of sampling technique, data collection tool, and methods used in the original studies to assess suicidal ideation.

**Table 1:** Characteristics of studies incorporated on the association between bullying victimization and suicidal ideation in Africa, 2024.

No.	Author and publication year	Country	Study design	School type	Sample size	Odds ratio	95% CI		Sampling technique	Data collection tool	Data collection methods	Quality status
							LL	UL				
1	Quarshie and Odame (58)	Ghana	CS	SS	1,101	0.96	0.69	1.32	RS	The Suicidal Behavior Questionnaire-Revised	SAQ	Low risk
2	Alabi et al. (59)	South Africa	CS	College	826	1.89	1.35	2.65	CS*	Asking the participants if they had ever experienced suicidal thoughts in their lives	SRQ	Low risk
3	Quarshie et al. (23)	Namibia	CS	SS	3,152	1.2	0.93	1.54	CASRS	2013 Namibia WHO-global student health survey	SA	Low risk
4	Aliy et al. (60)	Ethiopia	CS	SS	1,144	2.2	1.4	3.4	RS	WMH-CIDI questionnaire	SA	Low risk
5	Sendagala et al. (61)	Uganda	CS	SS	3,431	1.39	0.99	1.94	CS*	Did you ever seriously consider attempting suicide in the past 12 months?	Self-reported	Low risk
6	Quarshie et al. (10)	Eswatini	CS	SS	2,513	1.75	1.34	2.28	CS*	WHO school-based survey of suicidal ideation	SRQ	Low risk
7	Azasu et al. (24)	Ghana	CS	SS	3,632	1.58	1.23	2.03	CS*	WHO school-based survey of suicidal ideation	Self-reported measures	Low risk
8	Asante et al. (18)	Ghana	CS	SS	1984	1.68	1.38	2.2	CASRS	Global School based Student Health Survey (2012)	SAQ	Low risk
9	Quarshie et al. (62)	Liberia	CS	SS	2,744	1.91	1.45	2.51	CS*	Global School based Student Health Survey	SA	Low risk
10	Salifu and Yidana (63)	Ghana	CS	University	400	3.12	1.77	5.5	Systematic sampling	Suicidal ideation and attitudes scale	Through WhatsApp platforms	Low risk
11	Shayo and Lawala (17)	Tanzania	CS	Primary and SS	3,793	1.9	1.5	2.4	CS*	Global School based Student Health Survey	SAQ	Low risk
12	Asante et al. (16)	Sierra Leone	CS	SS	2,798	2.66	1.93	3.66	CASRS	Used data from the 2017 Sierra Leone WHO-GSHS	SAQ	Low risk
13	Okobi et al. (64)	Liberia	CS	SS	2,744	1.86	1.41	2.46	CS*	2017 Liberia Global School-based Health Survey	SAQ	Low risk
14	Baiden et al. (57)	Ghana	CS	SS	1,633	1.71	1.29	2.27	CS*	2012 Ghana Global School-Based Health Survey	SAQ	Low risk
15	Yedong et al. (76)	Mali	CS	SS and University	606	1.7	1.1	2.7	Convenience	Assessed by asking “Have you ever thought about taking your own life?”	SAQ	Low risk
16	Rudatsikira et al. (77)	Uganda	CS	SS	1,506	1.34	0.96	1.87	CS*	Global School-Based Health Survey-2003	SAQ	Low risk

CS, Cross sectional; UL, Upper Limit; LL, lower Limit; CS*, Cluster sampling; SAQ, Self-Administered Questionnaire; CASR, Cluster and simple random; RS, random sampling; SA, self-administered; SRQ, self-report questionnaire; SS, Secondary school.

### Meta-analysis

#### Pooled association between bullying victimization and suicidal ideation

**Figure 2:**
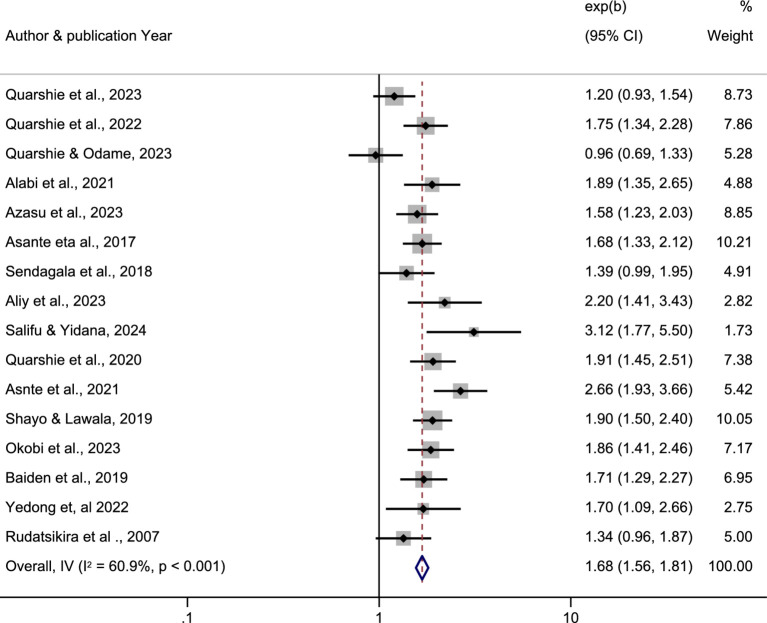
A forest plot showed the association between bullying victimization and suicidal ideation among students in Africa 2024.

The pooled estimated association between bullying victimization and suicidal ideation was calculated by pooling sixteen studies (10, 16–18, 23, 24, 57–59, 60–64, 76, 77) and found to be 1.68 (95% CI: 1.56–1.81; I^2^ = 60.9%, *p* < 0.001). The finding of this meta-analysis revealed that students who were victims of bullying were 1.7 times more fold to have suicidal ideation than non-bullying victimized students (Figure 2).

#### Heterogeneity and subgroup analysis

**Figure 3:**
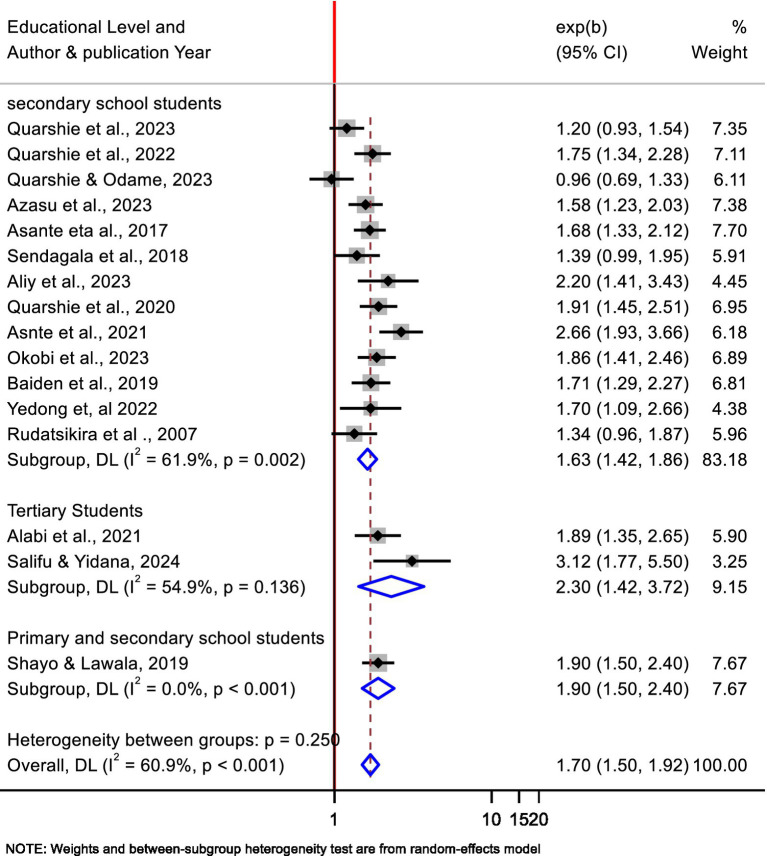
Subgroup analyses by educational level of participants for the pooled association between bullying victimization and suicidal ideation of students in Africa, 2024.

**Figure 4:**
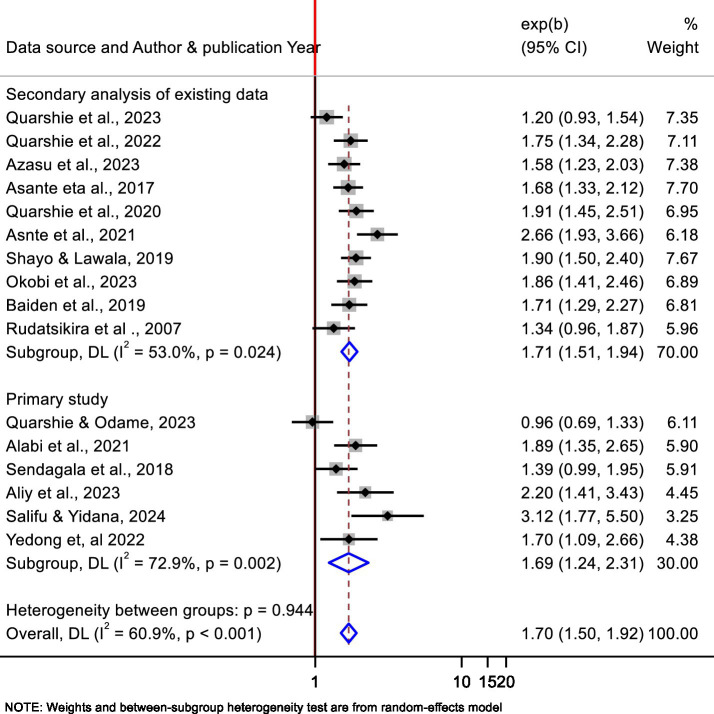
Subgroup analyses by data source for the pooled association between bullying victimization and suicidal ideation of students in Africa, 2024.

In this meta-analysis, a moderate heterogeneity was found among the included studies. To acknowledge this heterogeneity, we conducted a subgroup analysis by educational level (secondary school levels and tertiary, which includes university and college). Accordingly, the pooled association of bullying victimization and suicidal ideation was 2.3 (95% CI: 1.42, 3.72, *p* = 0.136, I^2^ = 54.9%) and 1.63 (95% CI: 1.42–1.86, *p* < 0.002, I^2^ = 61.9%) for tertiary and secondary school students, respectively (Figure 3). In addition, subgroup analysis by data source (using secondary data sources and conduct primary study) showed that 1.71 (95% CI: 1.51, 1.94, *p* = 0.024, I² = 53.0%) and 1.69 (95% CI: 1.24–2.31, *p* = 0.002, I² = 72.9%) for using secondary and primary data sources, respectively (Figure 4).

#### Publication bias

**Figure 5:**
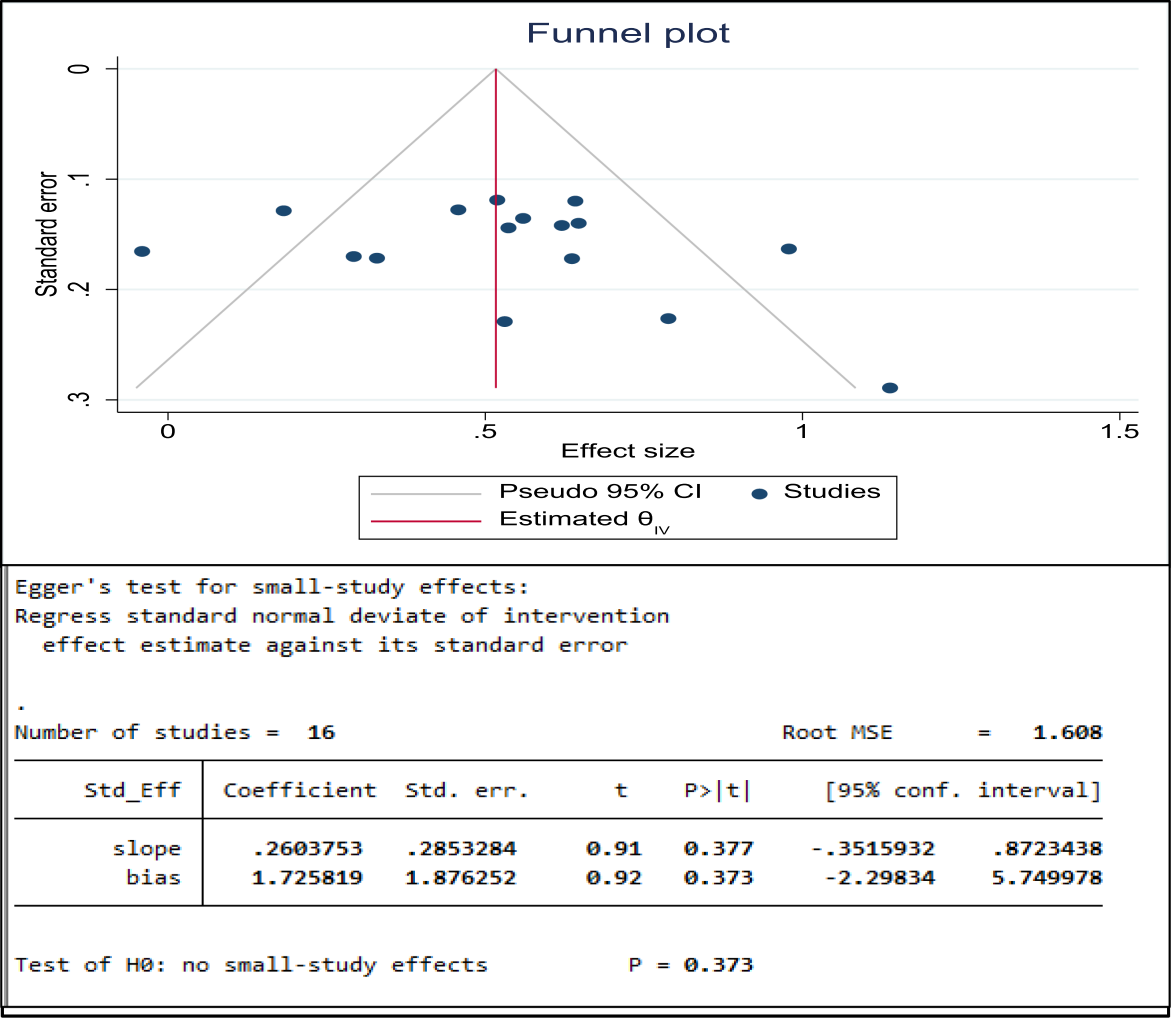
Funnel plot and Egger’s test for the association between bullying victimization and suicidal ideation of students in Africa, 2024.

The existence of publication bias was assessed within the included studies. Publication bias occurs when studies with noteworthy findings have a higher chance of being published than those with no significant results. To limit the risk of selective publication, a comprehensive literature search was conducted using various databases. Indeed, publication bias was evaluated using a Begg’s funnel plot and Egger’s regression test at a *p <* 0.05. Accordingly, the funnel plot for the estimated association of bullying victimization and suicidal ideation indicates that the distribution of studies was asymmetrical, and Egger’s test was found to be statistically insignificant (*p* = 0.373), meaning that there was no evidence of publication bias (Figure 5).

#### Sensitivity analysis

**Figure 6:**
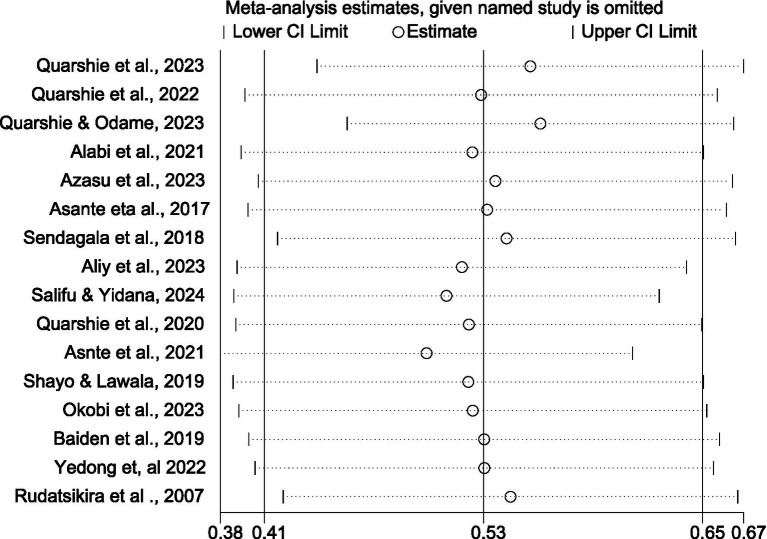
Sensitivity analysis graph to examine the effect of each study on the pooled result association between bullying victimization and suicidal ideation among students in Africa, 2024.

We performed a sensitivity analysis to evaluate the effect of each study on the pooled estimated associations between bullying victimization and suicidal ideation. We carefully excluded studies with risk of bias and examined how various measures of size impacted the overall results. The sensitivity analysis confirmed that the size remained consistent around 0.41 and 0.65, and none of the included papers seems to be an extreme outlier that affects the overall estimate significantly. The confidence intervals (CIs) for the majority of research overlapped with this value, and no single study appears to significantly affect the pooled estimate. While certain confidence intervals are wider than others, no studies appear to have a significantly disproportionate influence. This supports the integrity of the results (Figure 6).

## Discussion

This systematic review and meta-analysis were aimed at finding the pooled association between suicidal ideation and bullying victimization. By finding the pooled association this meta-analysis will provide robust evidence of the psychological and emotional toll bullying has for increasing the risk of suicidal ideation. As a result, the findings demonstrated that students with bullying victimization were at higher risk to have suicidal ideation than their counterparts who were not victimized. In addition, the subgroup analysis of this meta-analysis demonstrated that a slightly higher heterogeneity with significant variation comes from secondary school students than tertiary students. This variation may be due to secondary school adolescents being at a vital developmental stage that faces challenges marked by identity formation, peer pressure, and increased emotional fragility (78, 79). This may have an effect on their bullying and suicidal thoughts (80). Indeed, low availability of mental health services, like lack of effective anti-bullying policies and support systems, unfavorable school climate may open a door for bullying (81, 82). Some societal factors, such as normalizing violent behavior or stigmatizing mental health disorders, inappropriate teacher responses or lack of qualified professionals to address bullying, and lack of engagement in school activities, deter students from seeking assistance (81). In addition, the rise of social media and digital platforms has encouraged cyberbullying among high school students due to the use of computers and smartphones. Such behavior allows harassment to continue after school hours (33, 80). Moreover, differences in economic adversity, school culture, and school support systems may be a cause for this difference. Furthermore, the included studies may have differences in providing counseling services, teacher training on prevention of bullying, and presence of anti-bullying policies (83).

In addition, the subgroup analysis of this meta-analysis demonstrated that no significant heterogeneity exists among the individual studies from university and college students. But the heterogeneity was moderate and the pooled odds ratio was high. The possible explanation is that, despite universities providing anti-bullying and mental health resources, some students may not use them due to stigma or a lack of awareness, and those from marginalized backgrounds may be more vulnerable to bullying and suicidal ideation (84). Indeed, the intensive social dynamics of university contexts frequently produce tremendous pressure to fit in and perform academically, which can lead to feelings of isolation and inadequacy. Such emotions may further increase students’ susceptibility to bullying and suicidal ideation (85, 86). Furthermore, the shift to university life frequently involves greater freedom, leading some students to experiment with substances or engage in risky behaviors, and the availability of full internet connection and social media platforms may expose them to cyberbullying (87).

The other subgroup by data source showed that slightly higher heterogeneity was found from primary studies than studies that used secondary data analysis. This may be due to the global school-based student health survey using large, nationally representative samples, consistent sampling procedures, and standardized questionnaires that yield more stable estimates. Conversely, primary studies conducted in different contexts and using different methodologies may result in higher heterogeneity than secondary analysis.

The finding of this meta-analysis revealed that students who are victims of bullying were more prone to have suicidal ideation than non-victimized students. The pooled effect size of this comprehensive meta-analysis is in agreement with the previous meta-analysis (28, 47, 48, 88). The possible plausible explanation for this may be due to bullying triggering cascades of psychological, social, and biological events that can lead to suicidal ideation (89). Bullying, as a prelude to stress and trauma, can dramatically increase the incidence of suicide ideation (90). Indeed, exposure to bullying and the stress it causes impairs mood regulation, emotional reactions, and problem-solving skills (33). It causes students to impose intense emotional distress, withdraw from their social interactions, and feel rejected by their peers (33, 35). This leads them to deprive essential social support; low self-esteem, a negative self-concept, and stigmatization further intensify suicidal thoughts (91). In addition, due to the nature and effect of bullying, students may use maladaptive coping mechanisms such as substance abuse that worsen the emotional distress or self-harm that increase suicidal thoughts (28). Bullying reinforces genetic predispositions to mental health issues that may further increase susceptibility to suicidal ideation (92). On top of that, bullying frequently leaves victims feeling powerless, unable to flee the torture or seek aid, leading to feelings of hopelessness and helplessness. This sense of uncontrollability drives the conviction that there is no way out of their emotional misery, and this may lead them to perceive suicide as the only way to reclaim control or stop their suffering (93). Another possible reason is that students who are victims resulted in negative cognitive distortions, though victims internalized the harmful messages from bullies and developed a distorted self-image, low self-esteem, self-blame, social isolation, and withdrawal from peers (64). They frequently feel ashamed of their victimization and do not report it, afraid that others will regard them as weak or deserving of the abuse (94). This humiliation, along with social isolation, exacerbates feelings of worthlessness and despair that create feelings of unwantedness and increase suicidal behaviors (95). Furthermore, peer victimization causes low self-worth (96), low self-esteem (97), and feelings of hopelessness and loneliness (98), all of which increase the risk of suicidal thoughts (99).

## Conclusion

The findings of this study demonstrated that students with bullying victimization were at higher risk of suicidal ideation than their counterparts who were not victimized. Therefore, interventions focused at school-based anti-bullying programs, counseling services, parental involvement, and improving social and peer supports were advised to mitigate the effect of bullying and reduce suicidal ideation.

### Implication of the study

This systematic review and meta-analysis have a notable implication to address bullying and reduced suicidal ideation in school settings. By showing an association between bullying victimization and suicidal ideation, the study informs the need for intervention for bullying victimization and suicidal ideation. These results serve to protect vulnerable students and reduce future occurrences. Moreover, the study has substantial implications for improving school safety and suicide prevention strategies. Furthermore, the results inform the importance of culturally tailored interventions and call for longitudinal and interventional research in Africa, where context-specific data remain limited.

### Strengths and limitations of the study

This systematic review and meta-analysis is one of the few reviews that focused on a critical and timely issue. In addition, the study follows a systematic literature search and adherence to PRISMA guidelines. Furthermore, this review includes studies from a wide range of geographical locations that increase its generalizability. It adds to the growing body of literature by showing bullying victimization as a significant determinant for poor mental health. Despite its strengths, this has limitations. Due to the cross-sectional nature of included studies, the finding may limit causal inference between bullying victimization and suicidal ideation. The self-reported nature of the data may introduce reporting bias. Moreover, the findings may not fully capture the experiences of students in each nation in Africa due to the limited number of studies from each nation.

## Data Availability

The original contributions presented in the study are included in the article/Supplementary material, further inquiries can be directed to the corresponding author.

## Footnotes

^1^ https://www.stata.com
