# Supplementary material for: The association between bullying victimization and suicidal ideation among students in Africa: a systematic review and meta-analysis
Source: Front Public Health. 2025 Sep 9;13:1556211. doi: 10.3389/fpubh.2025.1556211 (PMC12455621; doi:10.3389/fpubh.2025.1556211)
Supplement: Supplementary file 1 [file Supplementary_file_1.docx]

Supplementary file 1

Search strategy, including keywords, synonyms, database, search date, and quantity of articles.

| Key words | Synonym | Data bases, combination, searching date and Number of articles found in each database | | | | |
| --- | --- | --- | --- | --- | --- | --- |
|  |  | PubMed and its combination | HINARI and its combination | Google scholar and Google | Semantic Scholar | Science direct |
|  |  | Date | Date | Date | Date |  |
|  |  | October 20/2024 | October 20/2024 | October 20/2024 | October 20/2024 | October 20/2024 |
|  |  | No of article **=2069** | No of article = 540 | No of article = 293 | No of article = 117 | No of article  93 |
| Bullying victimization | Bullying, peer victimization, school violent**,** cyber bullying | (((((((((((((((((((((((((((((((((((((((((((((((((((((((((((((((((((Bullying[Title/Abstract]) OR ("peer victimization"[Title/Abstract])) OR ("cyber bullying"[Title/Abstract])) OR ("school violence"[Title/Abstract])) AND (suicide[Title/Abstract])) OR ("suicidal thoughts"[Title/Abstract])) OR ("suicidal behavior" [Title/Abstract])) AND (Student*[Title/Abstract])) OR (adolescent*[Title/Abstract])) OR (youth[Title/Abstract])) OR ("school children"[Title/Abstract])) AND (Africa[Title/Abstract])) OR ("Sub-Saharan Africa"[Title/Abstract])) OR ("North Africa"[Title/Abstract])) OR (Algeria[Title/Abstract])) OR (Angola[Title/Abstract])) OR (Benin[Title/Abstract])) OR (Botswana[Title/Abstract])) OR ("Burkina Faso"[Title/Abstract])) OR (Burundi[Title/Abstract])) OR (Cameroon[Title/Abstract])) OR ("Cape Verde"[Title/Abstract])) OR ("Central African Republic"[Title/Abstract])) OR (Chad[Title/Abstract])) OR (Comoros[Title/Abstract])) OR (Congo[Title/Abstract])) OR ("Congo Brazzaville"[Title/Abstract])) OR ("Congo Kinshasa"[Title/Abstract])) OR ("Democratic Republic of the Congo"[Title/Abstract])) OR (Djibouti[Title/Abstract])) OR (Egypt[Title/Abstract])) OR ("Equatorial Guinea"[Title/Abstract])) OR (Eritrea[Title/Abstract])) OR (Eswatini[Title/Abstract])) OR (Ethiopia[Title/Abstract])) OR (Gabon[Title/Abstract])) OR (Gambia[Title/Abstract])) OR (Ghana[Title/Abstract])) OR (Guinea[Title/Abstract])) OR ("Ivory Coast"[Title/Abstract])) OR ("Cote d'Ivoire"[Title/Abstract])) OR (Kenya[Title/Abstract])) OR (Lesotho[Title/Abstract])) OR (Liberia[Title/Abstract])) OR (Libya[Title/Abstract])) OR (Madagascar[Title/Abstract])) OR (Malawi[Title/Abstract])) OR (Mali[Title/Abstract])) OR (Mauritania[Title/Abstract])) OR (Mauritius[Title/Abstract])) OR (Morocco[Title/Abstract])) OR (Mozambique[Title/Abstract])) OR (Namibia[Title/Abstract])) OR (Niger[Title/Abstract])) OR (Nigeria[Title/Abstract])) OR (Rwanda[Title/Abstract])) OR (Senegal[Title/Abstract])) OR (Seychelles[Title/Abstract])) OR ("Sierra Leone"[Title/Abstract])) OR (Somalia[Title/Abstract])) OR ("South Africa"[Title/Abstract])) OR ("South Sudan"[Title/Abstract])) OR (Sudan[Title/Abstract])) OR (Tanzania[Title/Abstract])) OR (Togo[Title/Abstract])) OR (Tunisia[Title/Abstract])) OR (Uganda[Title/Abstract])) OR (Zambia[Title/Abstract])) OR (Zimbabwe[Title/Abstract]) | ((Abstract:(bullying)) OR (Abstract:("peer victimization")) OR (Abstract:("school violence")) OR (Abstract:("cyber bullying")) OR (Abstract:("Suicidal Ideation")) OR (Abstract:(suicide)) OR (Abstract:("suicidal thoughts")) OR (Abstract:("suicidal behavior"))) AND ((Abstract:(Student*)) OR (Abstract:(adolescent*)) OR (Abstract:(youth))) AND ((Abstract:(Africa)) OR (Abstract:("sub-Saharan Africa")) OR (Abstract:(North Africa)) OR (Abstract:(Ethiopia)) OR (Abstract:("South Africa")) OR (Abstract:(Uganda)) OR (Abstract:(Tanzania)) OR (Abstract:(Nigeria)) OR (Abstract:(west Africa)))  **Filters applied**: Abstract, Free full text, Full text online, English | We apply direct search for Goggle scholar and Google, Web of science and science direct | | |
| Suicidal ideation | suicide, suicidal thought, suicidal behavior |  |  |  |  |  |
| Students | Adolescent, youth, school children |  |  |  |  |  |
| Africa | Sub-Saharan Africa, North Africa, and all African counties  , Algeria, Angola, Benin, Botswana, Burkina Faso, Burundi, Cameroon, Cape Verde", Central African Republic, Chad, Comoros, Congo, Congo Brazzaville, Democratic Republic of the Congo, Djibouti, Egypt, Equatorial Guinea, Eritrea, Eswatini, Ethiopia, Gabon, Gambia, Ghana, Guinea, Ivory Coast, Cote d'Ivoire, Kenya, Lesotho, Liberia, Libya, Madagascar, Malawi, Mali, Mauritania, Mauritius, Morocco, Mozambique, Namibia, Niger, Nigeria, Rwanda, Senegal, Seychelles, Sierra Leone, Somalia, South Africa, South Sudan, Sudan, Tanzania, Togo, Tunisia, Uganda, Zambia, Zimbabwe |  |  |  |  |  |
| Hint: Filters applied for PubMed: Abstract, Free full text, Full text, Observational Study, English, Humans. | | | | | | |
